# *In vitro* and *in vivo* inhibitory effects and transcriptional reactions of graphene oxide on *Verticillium dahliae*

**DOI:** 10.1128/spectrum.01276-25

**Published:** 2025-08-26

**Authors:** Ziqian Li, Xin Zhao, Mengmeng Jiang, Chenxing He, Ziyi Wang, Yunmei Zhang, Fuxin Wang, Shuling Zhang

**Affiliations:** 1 School of Life Sciences, Hebei University 162640 , Baoding, P. R. China; 2 Hebei Basic Science Center for Biotic Interaction, Hebei University 56667, Baoding, P. R. China; Connecticut Agricultural Experiment Station, New Haven, Connecticut, USA

**Keywords:** graphene oxide, membrane disruption, transcriptome analysis, *Verticillium dahliae*

## Abstract

The properties of graphene oxide (GO) against viruses and bacteria have received widespread attention, yet research into its antifungal capabilities remains comparatively scarce. Assessing the inhibitory effect of GO on plant pathogenic fungi is crucial for its application in preventing and managing fungal diseases. *Verticillium dahliae* represents a quintessential soil-borne fungal pathogen that infiltrates through plant roots and resides within the vascular tissues, leading to devastating wilt diseases. This research endeavored to explore the antimicrobial effect of GO on *V. dahliae* and to elucidate its underlying molecular mechanisms. Our findings revealed that GO exerted a significant inhibitory impact on mycelial growth and spore germination of *V. dahliae*. In addition, GO can significantly reduce the infection of *V. dahliae* on cotton. Biochemical analyses indicated that the DNA, RNA, malondialdehyde (MDA), and protein content within *V. dahliae* cells were markedly elevated post-GO treatment, underscoring the compromised cell membrane integrity. Transcriptome analysis revealed 1,149 differentially expressed genes (DEGs) during hyphal elongation and 1,078 DEGs in the process of spore germination. The downregulated genes predominantly encoded proteins involved in membrane integrity, such as intrinsic and integral membrane components, as well as proteins with oxidoreductase and transporter activities. Collectively, our data suggest that GO impedes mycelia growth and spore germination of *V. dahliae* by disrupting cell membrane integrity. This study offers novel perspectives for the development of antifungal agents and more precise clues to discover the mechanism by which GO suppresses the proliferation of *V. dahliae*.

**IMPORTANCE:**

GO could suppress the proliferation of the mycelia and the initiation of spore germination of *V. dahliae*. In addition, the utilization of GO could mitigate the manifestations of cotton wilt resulting from the infection by *V. dahliae*. This study offers novel perspectives for the development of antifungal agents and more precise clues to discover the mechanism by which GO inhibits the growth of *V. dahliae*.

## INTRODUCTION

Verticillium wilt, which is a ruinous fungal ailment triggered by diverse *Verticillium* strains, poses a significant threat to agriculture globally (1). Among the 10 species of *Verticillium*, *Verticillium dahliae* exhibits an extensive host range, infecting more than 200 host species globally on account of its pronounced pathogenicity (2–4). Typically, it initiates the invasion of plant roots, with its hyphae piercing through the root surface and colonizing the vascular bundles (5–7). This process ultimately leads to the death of the plant, exerting a profound negative impact on both the quality and yield of the plants, consequently giving rise to significant economic losses (5–7). The prevalent use of chemical fungicides to combat Verticillium wilt has raised concerns about potential resistance development and environmental contamination associated with their prolonged and excessive application. Therefore, it is urgent to explore a new and effective strategy for controlling Verticillium wilt triggered by *V. dahliae*.

Recently, the application of graphene, a new type of nanomaterial, has attracted widespread attention (8–10 Notably, graphene oxide (GO), which is a crucial derivative of graphene, is considered a potential candidate for antimicrobial agents due to its low toxicity to humans and the environment, and toxic effects on bacteria (11–13), fungi (14, 15), and plant pathogens (9, 16). Currently, research on graphene’s ability to suppress fungi, particularly those harmful to plants, is still in its infancy. The mycelial growth and spore germination of *Bipolaris sorokiniana* were both inhibited in a dose-dependent manner by GO treatment, and the addition of GO significantly alleviated the infection of pathogenic fungi in host plants (9). GO exhibits superior bactericidal effect even at extremely low doses in water (250 µg·mL^−1^), almost killing 94.48% *Xanthomonas oryzae* pv. *oryzae* (Xoo) cells (12). The germination of *Plasmopara vitricola* sporangia can be effectively inhibited by 50 µg·mL^−1^ GO-Fe_3_O_4_ nanocomposite material, and 250 µg·mL^−1^ GO-Fe_3_O_4_ on grape leaves in the field can significantly reduce the severity of downy mildew (16). Preliminary results have highlighted GO’s potential to exert strong antifungal effects. Despite the growing interest, scant knowledge exists regarding the research on the inhibitory effect of graphene against *V. dahliae*.

Many researches have been carried out to unravel the mechanisms underlying the antimicrobial activity of GO. Presently, two main antibacterial mechanisms of GO, including disrupting the cell membrane integrity and inducing oxidative stress, have been put forward (17, 18). Membrane disruption occurs when GO blankets and compromises the integrity of bacterial cell membranes, leading to cytoplasmic leakage or even cell lysis (12). A study has shown that upon exposure to GO, some conidia of *Fusobacterium oxysporum* and *Fusobacterium graminearum* underwent deformation and ceased germinating as a result of cell swelling and lysis, which reduced the vitality of conidia and prevented the initiation of infection cycles (19). Similarly, GO has been found to have a significant bactericidal effect on *Candida albicans*, as it causes cell membrane disruption, resulting in cell lysis (20). Oxidative stress is another mechanism of GO antibacterial activity, which involves the oxidation of fatty acids by lipid peroxides generated by reactive oxygen species (ROS) (21). These ROS can damage RNA and DNA and impair the ability of bacteria to sustain their regular physiological redox-regulated function, which eventually causes cell death (13). The application of GO to fungi has been shown to increase the production of ROS (22). In addition, GO has been documented to prompt alterations in the manifestation of genes associated with iron homeostasis, amino acid metabolism, and hunger and stress responses in *Saccharomyces cerevisiae* (23). However, scant reports exist regarding the molecular mechanisms of GO against plant pathogenic fungi.

RNA-seq, a pioneering technique in molecular biology, stands out for its precision and sequencing depth. It provides a detailed view of genome-wide changes, highlighting shifts in cellular functions and metabolic routes as organisms encounter varying environmental stressors. Particularly, this method has proven instrumental in examining the gene expression patterns of plant pathogens when subjected to fungicide exposure, revealing their genetic adaptability and reaction mechanisms (24–27). In this research, the inhibitory effects of GO regarding the multiplication of *V. dahliae* both under *in vitro* and *in vivo* conditions, and transcriptome analyses were used to elucidate the gene expression modulation exerted by GO on *V. dahliae*. These findings are instrumental in deepening our comprehension of GO’s antifungal efficacy and are anticipated to contribute valuable insights into the management strategies for Verticillium wilt disease triggered by *V. dahliae*.

## MATERIALS AND METHODS

### Fungal strain and materials

GO suspension was bought from Suzhou TANFENG graphene Tech Co. Ltd. Its purity is 99.9%, the layer diameter is 300–500 nm, the thickness is 0.335–1 nm, the number of layers is 1–2, the carbon content is 45%, the oxygen content is 54%, the sulfur content is 1%, and the ζ-potential is −34 mV. The highly virulent strain Vd991 of *V. dahliae* was maintained on potato dextrose agar (PDA) medium at 4°C. To prevent the reduction of strain viability, the aged cultures were subcultured onto a new slant every two months. For the fungal infection experiment, the cotton variety “Zhongzhimian 12” was employed.

### Effect of GO on mycelial growth of *V. dahliae*

The mycelia growth curve was carried out following the previously described standard protocols (28). *V. dahliae* strain Vd991 was inoculated onto PDA medium for 7 days at 25°C. Because our preliminary experimental results found that when the concentration of GO exceeded 200 µg·mL^−1^, it would inhibit the seeds’ germination rate and growth and development of cotton (the data are currently being prepared for publication in another paper). Therefore, a 1 cm diameter mycelial piece was positioned on PDA medium containing 0 (CK), 50, 100, and 200 µg·mL^−1^ GO at 25℃. The colony diameter of each treatment was measured every other day at the same time, and the growth curve was acquired following 8 days of incubation.

The mycelial biomass of the *V. dahliae* was further determined. Briefly, three 1 cm diameter pieces of activated mycelia were placed into 300 mL Czapek medium with 0, 50, 100, and 200 µg·mL^−1^ GO in conical flasks. Post-shaking culture at 180 rpm and 25°C for 4 days, the mycelia were harvested by filtration and rinsed with distilled water thrice. The dry weight of the mycelium was measured after being dried to a constant weight at 50°C. Each experiment was conducted under a completely randomized design with three repetitions.

### Effect of GO on spore growth of *V. dahliae*

After culturing on PDA medium at 25°C for 7 days, 1 cm diameter activated mycelia were transferred to fresh PDA medium and cultured for 12 days under dark conditions. The mycelia were then rinsed with 0, 50, 100, and 200 µg·mL⁻¹ GO solutions, respectively. Subsequently, 15 µL of spore suspension was dropped onto a concave slide for incubation in the dark. The number of germinated spores and the length of germ tubes were observed under a microscope at 10, 20, and 24 hours of incubation. The length of the spore tube is greater than the short radius of the spore; it is considered to have germinated. For each treatment, three concave slides were utilized, and at least 100 spores were examined. The spore germination rate was computed by means of the following formula:

Spore germination rate (%) = (number of germinated spores/total number of spores) × 100%.

### Structural and morphological characterization by scanning electron microscopy

The effect of GO on the morphology of the mycelium of *V. dahliae* was observed using scanning electron microscopy (SEM, JSM-IT500). The mycelium with a diameter of 1 cm, which had been cultured on PDA for 7 days, was transferred to Czapek medium containing 0 and 200 µg·mL^−1^ GO, respectively, and cultured for 4 days under shaking (180 r·min^−1^). Then, the mycelium was collected from the medium and fixed overnight at 4°C with 2.5% glutaraldehyde. The fixed samples were rinsed with phosphate-buffered solution (PBS, pH 7.0) for 15 minutes and repeated three times. Then, the samples were dehydrated successively in ethanol solutions with concentrations of 30%, 50%, 70%, 80%, 90%, and 95% for 15 minutes per stage, and finally dehydrated in anhydrous ethanol for 20 minutes. The dehydrated samples were observed under the scanning electron microscope after vacuum freeze-drying and gold sputtering coating.

### Measurement of the chitin content

After culturing on PDA medium at 25°C for 7 days, the mycelium was transferred to Czapek medium containing 0 and 200 µg·mL^−1^ GO and shaken culture for 4 days (180 r·min^−1^). The mycelium was collected, ground into a white powder with liquid nitrogen, dissolved in water, disrupted for cell walls using an ultrasonic crusher, and then lyophilized. 70 mg of lyophilized powder was weighed, 6 mL of 6M hydrochloric acid was added, reacted at 90°C for 12 hours, and evaporated at 50°C to remove hydrochloric acid. The evaporated sample was dissolved in an equal volume of water, centrifuged, 1 mL of the supernatant was taken, and 0.25 mL of 4% (vol/vol) acetylacetone solution was added and heated in a 90°C water bath for 1 hour. Then, 2 mL of ethanol and 0.25 mL of Ehrlich’s reagent were added, mixed well, and the OD at 540 nm was measured.

### Measurement of cell membrane integrity

Fluorescein diacetate (FDA)/propidium iodide (PI) dual color fluorescence method was used to measure cell membrane integrity. 100 mg of FDA (Solarbio, Beijing, China) was dissolved in 4 mL of acetone to prepare a 25 mg·mL^−1^ mother liquor, which was stored in a brown bottle at −20°C. For application, the FDA storage solution was diluted to obtain 100 µg·mL^−1^ FDA staining solution. 0.01 g of PI was weighed and dissolved in 10 mL of 0.01 mol·L^−1^ PBS (pH 7.4) buffer to prepare a stock liquor with a concentration of 1 mg·mL^−1^, which was stored in a brown bottle at 4°C away from light. Subsequently, it was diluted with PBS buffer to obtain a 3 µg·mL⁻¹ staining solution for further use. After culturing in Czapek medium containing 0 and 200 µg·mL⁻¹ GO for 4 days, spore suspensions were collected and incubated with FDA staining solution at a final concentration of 100 µg·mL⁻¹ in the dark at 25°C for 20 minutes. They were then incubated with PI staining solution having a final concentration of 60 µg·mL^−1^ in the dark at 4°C for 10 minutes. Spores were observed under a fluorescence microscope with excitation and emission wavelengths of 488 nm and 630 nm, respectively.

### Assessment of the leaked DNA, RNA, and protein

Macromolecular substances like DNA, RNA, and protein are discharged from cells with disrupted cell membranes. Equivalent mycelia of *V. dahliae*, which had been incubated on PDA medium at 25°C for 7 days, were transferred to Czapek medium with 0 and 200 µg·mL^−1^ GO, respectively, and cultivated for 4 days with shaking (180 r ·min^−1^). The contents of DNA and RNA in the medium were detected by UV absorption at 260 nm, while the protein content in the medium could be measured by UV absorption at 280 nm.

### Measurement of the relative permeability and MDA

*V. dahliae* mycelium (5 g) cultured on PDA for 7 days was rinsed with distilled water and then transferred to 50 mL of distilled water and 200 µg·mL^−1^ GO solution separately. The conductivity was measured after being left for 15, 30, 60, 90, 120, and 180 minutes. The samples were then boiled in water for 10 minutes, and conductivity was measured again. The relative permeability was calculated. Relative permeability = (C − C_0_)/(C kill treatment − C_0_) ×100% (C is the conductivity after treatment; C_0_ is the conductivity at the beginning; C kill treatment is the conductivity that kills the mycelium).

After *V. dahliae* was shake-cultured for 4 days in Czapek medium containing 0 and 200 µg·mL⁻¹ GO, respectively, 0.2 g mycelium was weighed and placed in a precooled mortar. 2 mL of 10% trichloroacetic acid (TCA) along with a bit of quartz sand was added and ground completely in an ice bath. Subsequently, 6 mL of 10% trichloroacetic acid was incorporated for further grinding. And then the mixture was centrifuged at 4°C and 10,000 rpm for 10 minutes using a high-speed refrigerated centrifuge. The supernatant obtained and culture medium were used to analyze the content of malondialdehyde (MDA) with MDA assay kit (Nanjing Jiancheng Bioengineering Co., Ltd., China).

### *V. dahliae* cells exposure to GO

**Fig 1:**
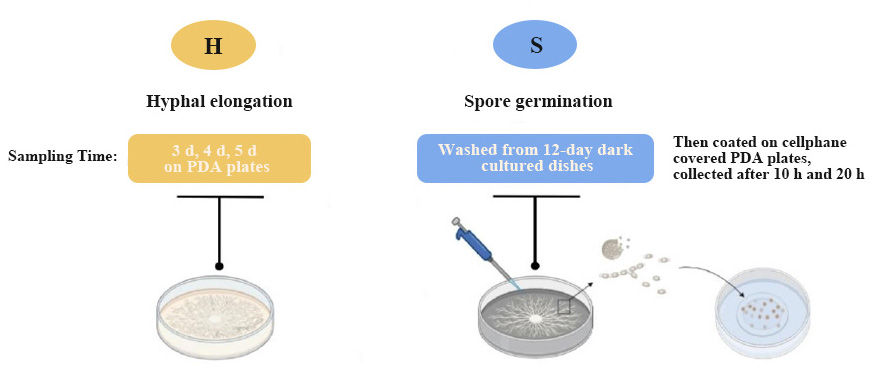
Schematic diagram of *V. dahliae* sampling.

The *V. dahliae* was cultured in PDA with 0 and 200 µg·mL^−1^ GO at 25°C. Sample collection details are shown in Fig. 1. Briefly, during light cultivation, the mycelia of *V. dahliae* that had grown on the surface of the PDA for 3, 4, and 5 days were harvested and uniformly blended to constitute the hyphal elongation period (H) sample. The mycelia that had been cultured in PDA at 25°C for 12 days under dark conditions were rinsed either with sterile water or 200 µg·mL^−1^ GO, and the spore suspension was aseptically transferred onto new PDA medium overlaid with sterile glassine paper. The spore suspension cultured for 10 h and 20 h was combined in equal volumes to form one spore germination period (S) sample. For every period and treatment, three biological replicates were collected and chilled in liquid nitrogen for 1 hour, after which they were stored at −80°C for RNA extraction.

### Transcriptome analysis of *V. dahliae*

Total RNA was isolated from the mycelia and spores of *V. dahliae* following the protocol of TRIzol Reagent. The purity and integrity of RNA were assessed by ND-2000 (NanoDrop Technologies) and agarose gel electrophoresis, respectively. Total RNA was sent to Majorbio Bio-pharm Biotechnology Co (Shanghai, China) for sample preparation and sequencing. Genes that were differentially expressed (DEGs) between the control and treatment groups were evaluated using DESeq2 (30). DEGs were defined as genes with |log2 (fold change) | ≥1 and a false discovery rate (FDR) significance score (adjusted *P*-value) <0.05. The Gene Ontology (GO) and Kyoto Encyclopedia of Genes and Genomes (KEGG) annotations for *V. dahliae* genes were identified using EggNOG-Mapper software (http://eggnog-mapper.embl.de/).

### Real-Time fluorescence quantitative PCR validation

Eight related genes were randomly selected from the transcriptome data for quantitative real-time PCR (qRT-PCR) validation. Primers were designed using Primer Premier 5.0 software (Table S1). The cDNA was synthesized using RNA as a template. qRT-PCR was performed using a Fast Start Universal SYBR Green Master Mix (Roche, USA). The qRT-PCR procedure was as follows: predenaturation at 95°C for 120 s, denaturation at 95°C for 5 s, annealing at 54°C for 30 s, and extension at 72°C for 30 s for 45 cycles. The β-tubulin gene was taken as the internal reference gene. The method of 2^-ΔΔCt^ was used to calculate the gene expression according to the Ct method (29).

### Effect of GO in preventing *V. dahliae* infection

Colonies of *V. dahliae* grown on PDA were cultured in Czapek medium for 7 days at 25°C with shaking (180 r·min⁻¹). Spore suspensions (10^7^ conidia·mL⁻¹) were adjusted with distilled water before inoculation. The seeds of cotton were put into a plastic beaker containing 250 mL of water and incubated at 28°C in the incubator. The germinated seeds were cultivated in a pot with a mixture (1:1 ratio, vol/vol) of nutrient soil and vermiculite and cultured at 28°C and 60% relative humidity with a 16/8 h light/dark cycle in the incubator. To assess the effect of GO in preventing *V. dahliae* infection, cotton seedlings with one true leaf were used for root injury inoculation experiments (30). The cotton seedlings, watered with either 200 mL of H_2_O (control) or 200 µg·mL⁻¹ GO solution, were inoculated with 10 mL 1 × 10^7^ conidia·mL⁻¹ conidia suspensions into the matrix near the injured cotton roots (31). The hypocotyls (1.5 cm upwards from the bottom of cotton seedlings) were collected from 10 plants in each treatment 2, 5, and 7 days after inoculation with *V. dahliae*, respectively. The hypocotyl section was disinfected in a 75% alcohol solution for 3 minutes, rinsed three times with sterile water, soaked in 10% H_2_O_2_ for 45 minutes, rinsed three times with sterile water, then cut into small sections and laid on PDA for incubation at 25°C. Part of the hypocotyls was used to measure *V. dahliae* biomass via qPCR. The fungus-specific primers (ITS1-F and ST-Ve1-R) and cotton *GhUB7* primers (endogenous control) are listed in Table S1. The disease index of the remaining cotton seedlings was investigated 21 days after inoculation.

### Data analysis

One-way ANOVA was performed using IBM SPSS Statistics (version 21.0, IBM Corp., Armonk, New York, USA) (*P < 0.05; **P < 0.01). Origin 2021 (version 8.5) software was used for plotting. The results are shown as means ± standard deviations (SD) from three biological replicates.

## RESULTS

### Effects of GO on mycelial growth and spore germination of *V. dahliae*

**Fig 2:**
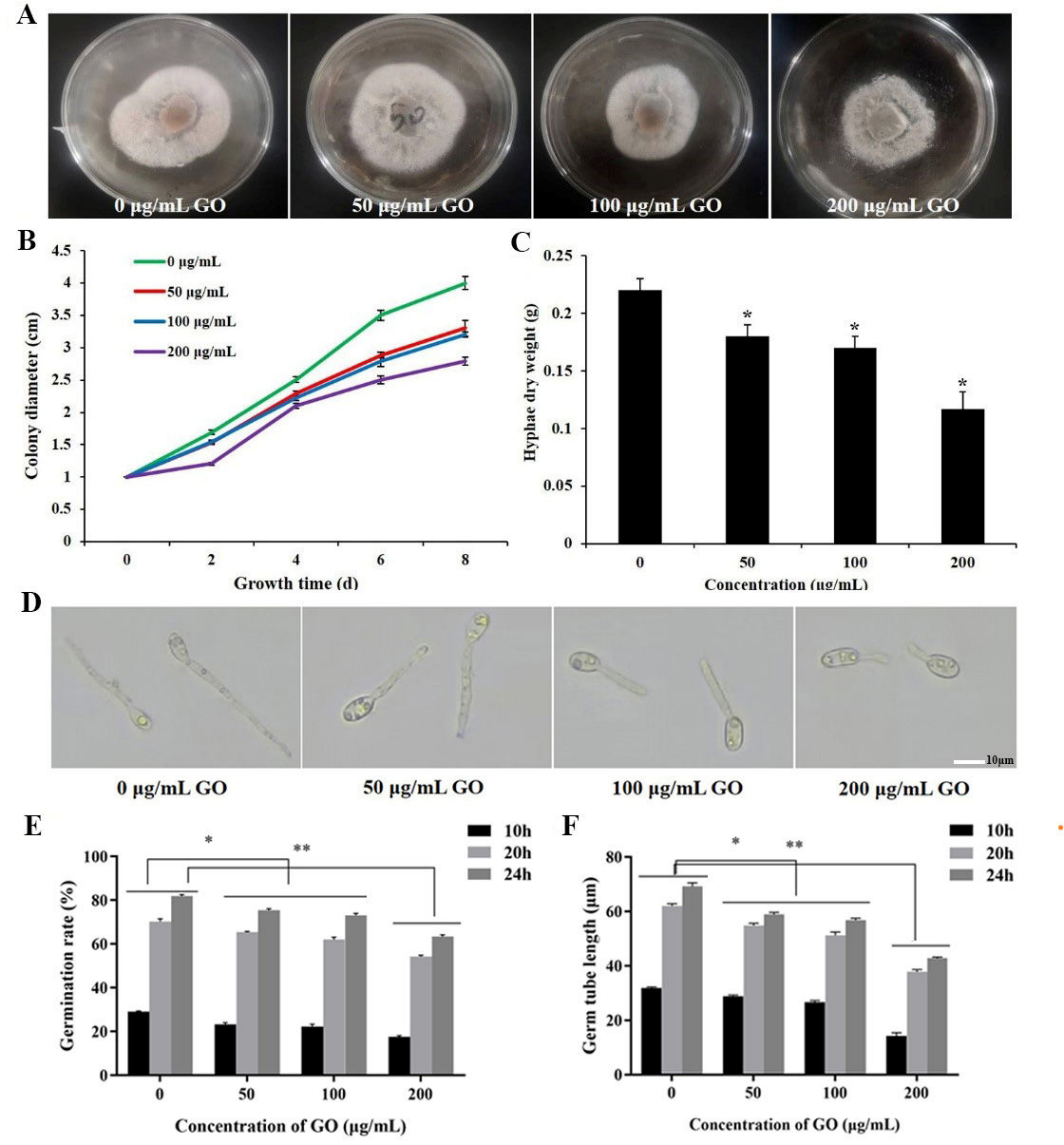
Growth inhibition of GO on mycelium and spore germination of *V. dahliae*. (**A**) The colony morphology of *V. dahliae* after 8 days of cultivation in PDA plates with various concentrations of GO. (**B**) The growth rate of *V. dahliae* mycelium on solid PDA plates with varying GO concentrations. Bars represent the standard errors (*n* = 3). (**C**) The dry weight of hyphae in Czapek medium under diverse GO concentrations for 4 days. (**D**) *V. dahliae* spore germination morphology upon treatment with different GO levels. (**E**) Spore germination rate of *V. dahliae* under different concentrations of GO treatment. (**F**) Germ tube length of *V. dahliae* under different concentrations of GO treatment. *P < 0.05, **P < 0.01.

Mycelium is an infectious structure that invades plant tissues and vascular systems, causing systemic plant infections (32). To examine the antifungal activity of GO against *V. dahliae*, the growth curve and hyphae dry weight exposed to various concentrations of GO (0, 50, 100, 200 µg·mL^−1^) were measured. As shown in Fig. 2A and B and Table S2, GO restrains the mycelial growth rate of *V. dahliae,* and the inhibitory effect is strengthened with increases in GO concentration in solid PDA media. After 8 days of treatment with 200 µg·mL^−1^ GO, the colony diameter and the hyphae density of *V. dahliae* obviously decreased compared to the control. To further demonstrate the inhibitory effects of GO on mycelial growth, the mycelial biomass of *V. dahliae* cultured in Czapek medium was measured after being treated with different concentrations of GO. Consistent with the results of culturing on PDA media, the mycelial biomass of *V. dahliae* declined with an increase in GO concentration, and the dry weights of mycelia were significantly decreased by 28%, 36%, and 52%, respectively (Fig. 2C).

Spore germination of filamentous fungi represents the initial essential stage in spread preparation (33). To enhance the understanding of GO’s antifungal properties, the effects of different concentrations of GO on *V. dahliae* spore germination were analyzed (Fig. 2D). Although the difference between the 50 and 100 µg·mL^−1^ GO treatments was not significant, overall GO exhibited a dose-dependent decrease in spore germination and germ tube elongation. Among them, the spore germination rates and the average length of the germ tubes at 10, 20, and 24 hours after treatment with 200 µg·mL^−1^ GO were 17.6%, 53.8%, 63.5% and 14.5, 37.2, 42.4 µm, respectively, all of which were extremely significantly lower than the control group’s 29.0%, 70.1%, 81.7% and 31.6, 61.4, 69.5 µm (Fig. 2E and F).

### Effects of GO on morphological alterations, cell membrane integrity, permeability, and lipid peroxidation of *V. dahliae*

**Fig 3:**
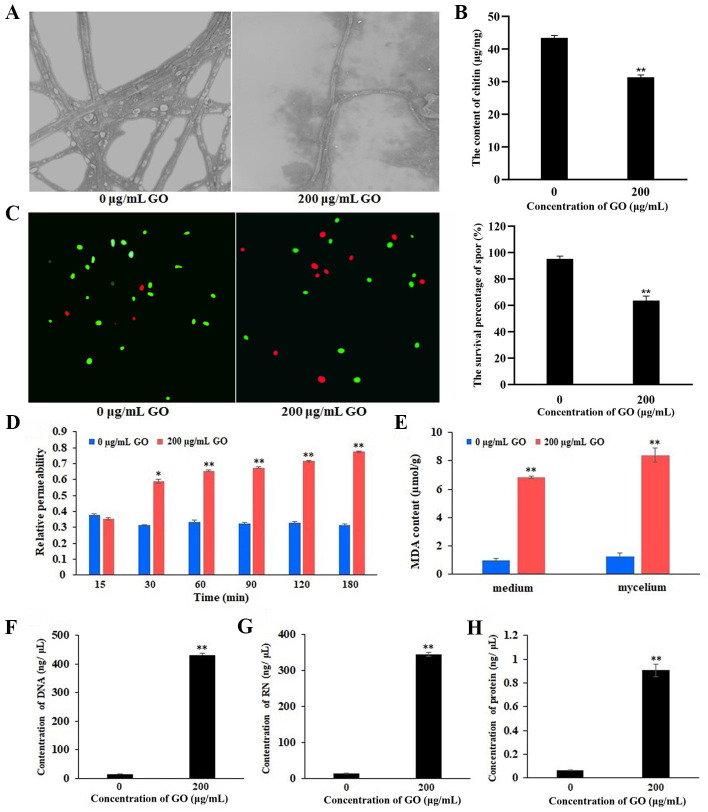
Inhibitory activities of GO on the cell wall and membrane system of *V. dahliae*. (**A**) Morphological structural alterations of *V. dahliae* mycelia induced by GO. (**B**) GO induced the change of chitin content in mycelium. (**C**) Spores unexposed to GO and exposed to GO of *V. dahliae* with FDA-PI double staining and quantity statistics. (**D**) The relative permeability changes in mycelium induced by GO. (**E**) The content of MDA in the medium and mycelium. (**F**, **G**, and **H**) DNA, RNA, and protein concentration in medium. *P < 0.05, **P < 0.01.

To investigate the antifungal mechanism of GO against *V. dahliae*, the morphology and structure of its mycelia were examined via SEM. As depicted in Fig. 3A, the mycelia of normal *V. dahliae* showed regular and uniform morphology, possessing smooth external surfaces and an obvious segmented appearance. Upon being treated with 200 µg·mL^−1^ GO, the mycelia of *V. dahliae* appeared thinner compared to the control, with unsmooth and irregular segmented cells. In addition, the chitin content of mycelia treated with 200 µg·mL^−1^ GO was significantly lower than that of the control group (Fig. 3B). Chitin, a crucial component of the fungal cell wall, plays a pivotal role in maintaining fungal cell structure, physiological functions, and interactions with the external environment. Therefore, it is speculated that the change in chitin content may be one of the causes of the morphological alteration of mycelia.

To further investigate whether the membrane integrity of *V. dahliae* was impaired by GO, we measured the integrity of the spore membrane using the FDA/PI dual-color fluorescence method. When the cell membrane is intact, PI cannot enter the cell, while FDA can enter the cell and produce fluorescent products by reacting with intracellular lipases. These fluorescent products accumulate intracellularly, emitting green fluorescence under blue light excitation. When the cell membrane is damaged, fluorescein cannot accumulate within the cell, preventing it from emitting fluorescence. PI can enter cells through damaged cell membranes, causing cells to emit red fluorescence. As shown in Fig. 3C, the number of spores emitting green fluorescence in the GO-treated group was significantly lower, while the number of spores emitting red fluorescence was notably higher, than that in the group without GO exposure.

When the cell membrane is damaged, the membrane permeability increases and important substances in the cytoplasm leak out. To verify this hypothesis, relative permeability and the efflux of cellular constituents, including DNA, RNA, protein, and malondialdehyde (MDA) content, were measured. The results suggested that the relative permeability of *V. dahliae* unexposed to GO remained basically unchanged, while the treatment group showed a significant increase after 30 minutes of treatment with 200 µg·mL^−1^ GO (Fig. 3D). Similarly, the MDA content in both the medium and mycelium is very low, while the MDA content in both increased significantly after treatment with 200 µg·mL^−1^ GO (Fig. 3E). In addition, the leakage of DNA and RNA leakage in the filtrate of spore suspensions treated with 200 µg·mL^−1^ GO was markedly greater than that in the group without GO treatment. The DNA concentration in the medium with 200 µg·mL^−1^ GO was 431.57 ng·µL^−1^, significantly higher than the DNA concentration (11.32 ng·µL^−1^) in the control group (Fig. 3F). Similarly, the RNA and protein concentrations in the medium containing 200 µg·mL^−1^ GO were 345.44 ng·µL^−1^ and 0.91 ng·µL^−1^, respectively, significantly higher than the control group’s 13.4 ng·µL^−1^ and 0.064 ng·µL^−1^ (Fig. 3G and H). These results indicated that GO damaged the membrane permeability of *V. dahliae*, leading to the leakage of DNA, RNA, protein, and MDA, as well as an increase in relative permeability.

### Evaluation of total differential expression genes

To evaluate gene expression modulation in *V. dahliae* associated with GO treatment, transcriptome sequencing was performed on samples collected from mycelia and spores treated with GO, as well as the non-treated control group. The primary quality metrics of RNA-seq data and complete information for each sample are presented in Table S3. In this study, approximately 42–56 million raw readings were obtained for each sample through Illumina high-throughput sequencing. After strict data quality control, all clean reads have high Q20 (>98%), Q30 (>95%), and GC (>57%). These clean reads, which could be mapped to the reference genome of *V. dahliae* (GCF_000150675.1; https://www.ncbi.nlm.nih.gov/genome/?term=Verticillium+dahliae), ranged between 88.25% and 91.49%, and the uniquely mapping portion of each sample was 87.24%–91.09%.

**Fig 4:**
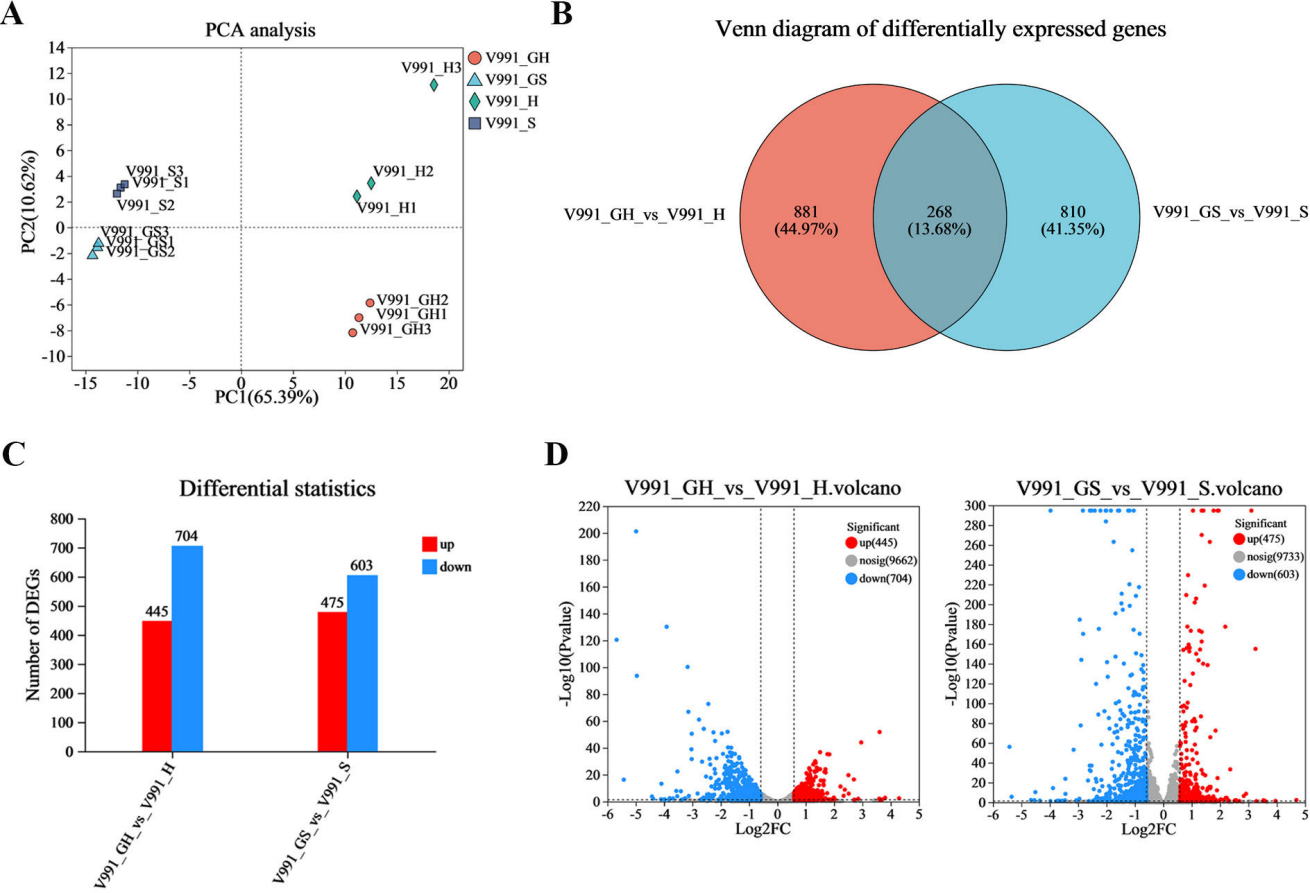
Transcriptomic analysis of mycelial and spore of *V. dahliae* treated with GO. (**A**) PCA of the four sample groups. (**B**) Venn diagram of differential genes among different treatments. (**C**) Histogram of DEGs number, red for upregulated genes, blue for downregulated genes, with the number on bars showing the gene quantity. (**D**) Volcano plot of gene expression differences among different groups and represent.

Principal component analysis (PCA) was used to evaluate transcriptomic variation among samples (Fig. 4A). The PCA plot showed that biological replicate samples are obviously clustered together. Samples of V991_S (non-exposure to GO) and V991_GS (exposure to 200 µg·mL^−1^ GO) clustered more closely than samples of V991_H (non-exposure to GO) and V991_GH (exposure to 200 µg·mL^−1^ GO). This indicated that the H period exhibited a much stronger transcriptomic response to GO exposure than the S period.

In the V991_GS vs V991_S and V991_GH vs V991_H groups, altogether 2227 DEGs were detected, and among these DEGs, 268 genes were shared between both groups (Fig. 4B). In V991_GH vs V991_H group and V991_GS vs V991_S group, DEGs were detected consisting of 445 upregulated, 704 downregulated genes and 475 upregulated, 603 downregulated genes, respectively (Fig. 4C). The volcano plot data indicated no significant difference in the overall number of upregulated and downregulated genes between V991_GS vs V991_S and V991_GH vs V991_H group, though the distribution of DEGs in the plot differed (Fig. 4D). This indicates that *V. dahliae* exhibits specific transcriptional responses to GO at different developmental stages.

### Enrichment analysis of GO

**Fig 5:**
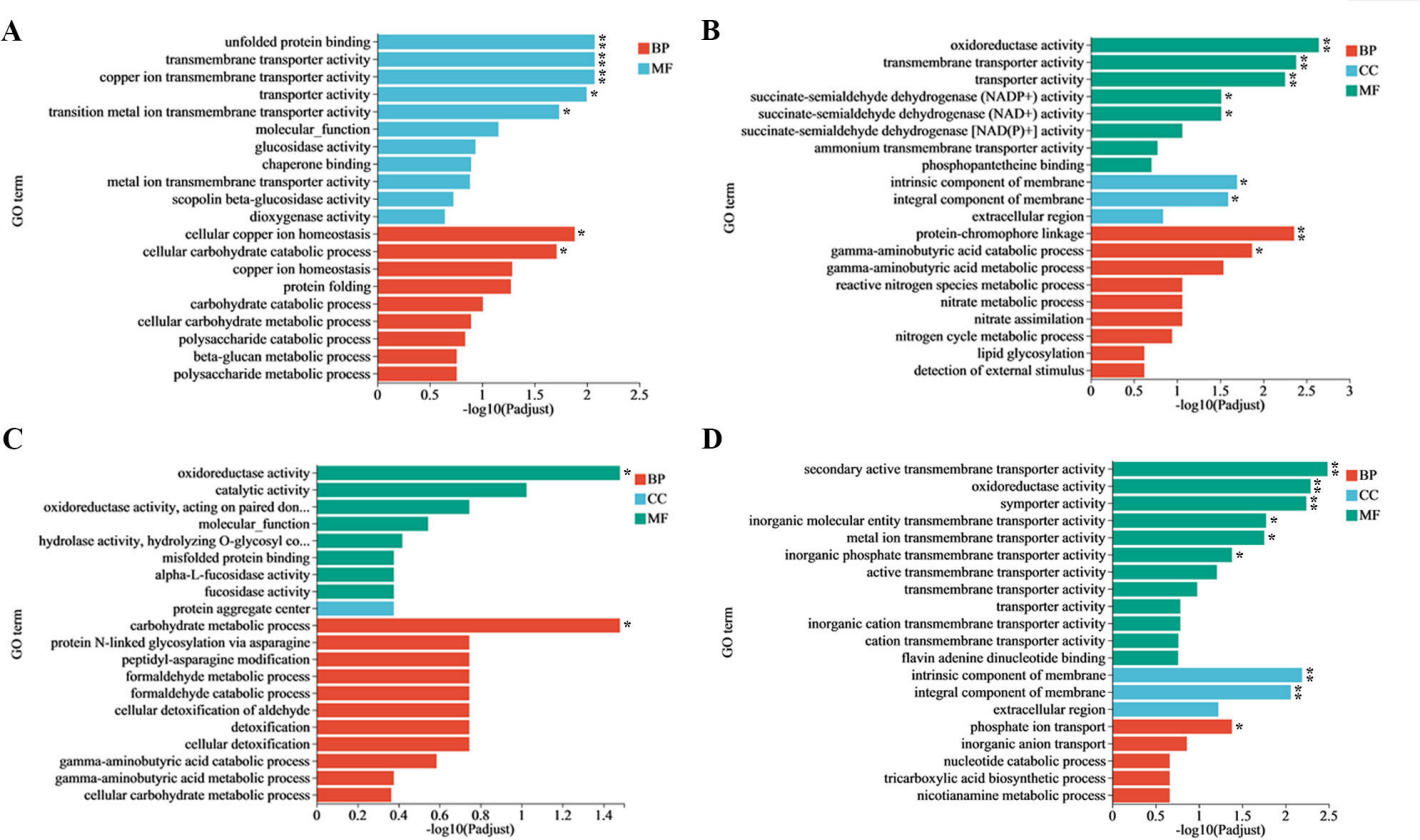
Secondary bar chart of up-GO terms (**A**) and down-GO terms (**B**) in V991_GH vs V991_H and up-GO terms (**C**) and down-GO terms (**D**) in V991_GS vs V991_S mapped by GO functional classification. The y-axis showed GO categories, namely biological process (BP), cellular component (CC), and molecular function (MF). The x-axis represented the degree of statistical difference. The symbol “**” in the figure denoted that the enriched GO term had a *P*adj <0.01, * *P*adj <0.05.

GO term enrichment analysis was performed separately for upregulated and downregulated DEGs to discover the biological functions altered when *V. dahliae* was treated with GO. The GO enrichment analysis indicated that the DEGs were grouped into three classes, namely molecular function (MF), cellular component (CC), and biological process (BP). In the V991_GH vs V991_H group, the upregulated DEGs were enriched in biological process (BP) and molecular function (MF) (Fig. 5A). The downregulated DEGs from the V991_GH vs V991_H group, upregulated and downregulated DEGs from the V991_GS vs V991_S group were enriched in BP, MF, and CC (Fig. 5B through D). Most notably, the down-categories from V991_GH vs V991_H and V991_GS vs V991_S groups were all significantly enriched in “intrinsic component of membrane” and “integral component of membrane” according to the CC categories. By the MF categories, the up-categories from V991_GH vs V991_H group were mainly classified into unfolded protein binding, transmembrane transporter activity, copper ion transmembrane transporter activity, transporter activity, and cellular copper ion homeostasis. Five down-categories (MF) related to metabolic processes were enriched, namely oxidoreductase activity, transmembrane transporter activity, transporter activity, succinate-semialdehyde dehydrogenase (NADP^+^) activity, and succinate-semialdehyde dehydrogenase (NAD^+^) activity. For the V991_GS vs V991_S group, only oxidoreductase activity of up-categories (MF) associated with metabolic processes enriched was statistically different, while six down-categories (MF) associated with metabolic processes were enriched, involving secondary active transmembrane transporter activity, oxidoreductase activity, symporter activity, inorganic molecular entity transmembrane transporter activity, metal ion transmembrane transporter activity, and inorganic phosphate transmembrane transporter activity.

### Enrichment analysis of DEGs in KEGG pathway

**Fig 6:**
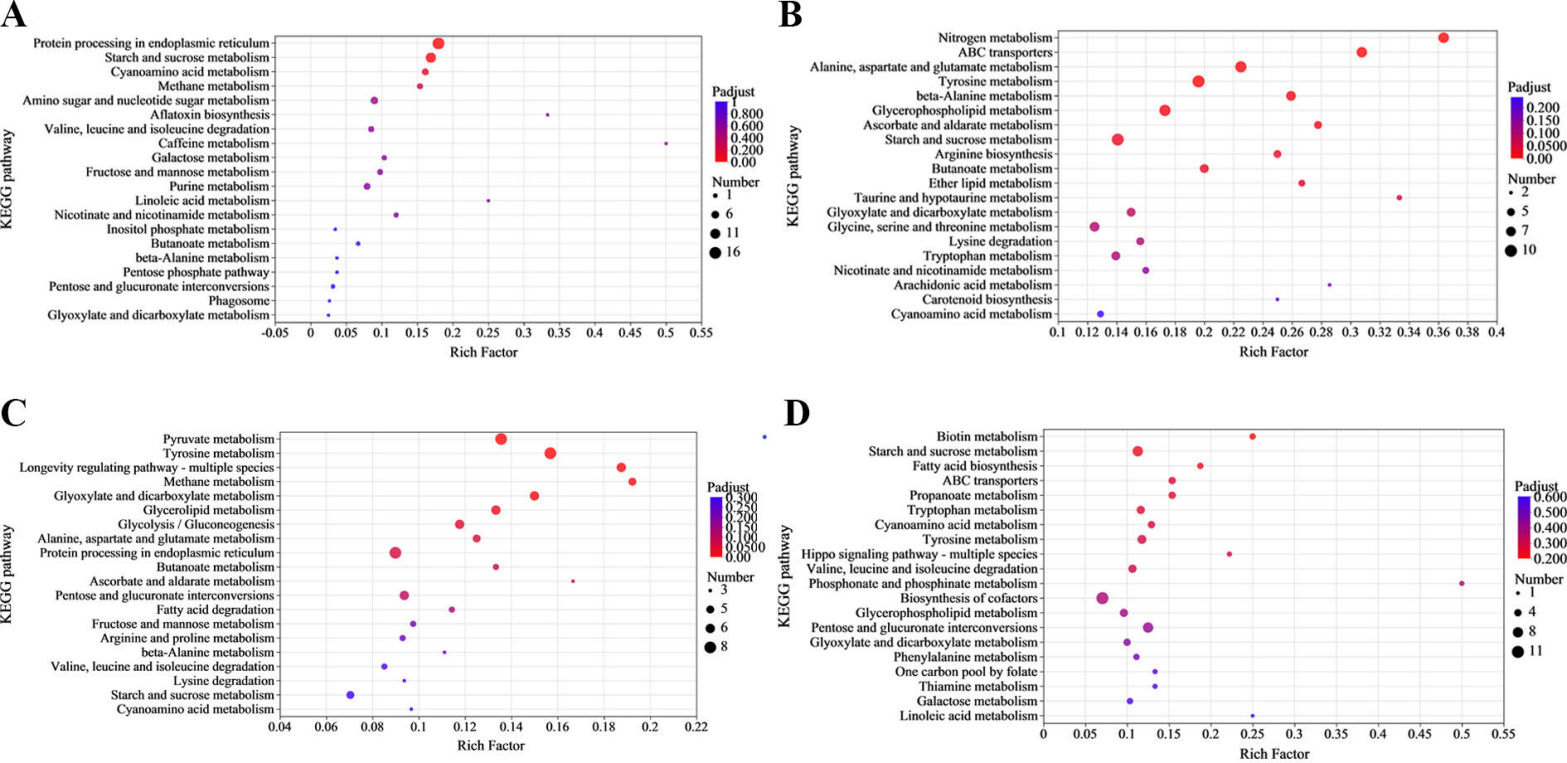
KEGG pathway enrichment analysis of upregulated (**A**) and downregulated (**B**) DEGs in V991_GH vs V991_H, and upregulated (**C**) and downregulated (**D**) in V991_GS vs V991_S. The y-axis presents the pathway name; the x-axis displays the gene ratio (rich factor) within each pathway. The bubble size stands for the quantity of differentially expressed genes. The gradient bar ranging from red to blue denotes the decrease of log10 (*P*-adjust).

KEGG analysis was used to elucidate concentrated biochemical pathways of DEGs. In the V991_GH vs V991_H group, the upregulated DEGs participated in genetic information processing and metabolism. They were prominently concentrated in protein processing within the endoplasmic reticulum and starch and sucrose metabolism, corresponding to folding, sorting, degradation, and carbohydrate metabolism, respectively (*P*adj <0.05) (Fig. 6A). Numerous downregulated DEGs responsible for carbohydrate and amino acid metabolism were influenced by GO. Three amino acid metabolism pathways and three carbohydrate metabolism pathways were significantly suppressed by GO (*P*adj <0.05) (Fig. 6B). In V991_GS vs V991_S group’s up-pathways, the overexpressed DEGs were chiefly enriched in diverse metabolic processes (pyruvate metabolism, tyrosine metabolism, longevity regulating pathway—multiple species, methane metabolism, glyoxylate and dicarboxylate metabolism, and glycerolipid metabolism) (Fig. 6C and D). In addition, ABC transporters related to fungal resistance and tyrosine metabolism associated with pathogenicity of fungi were all enriched in V991_GH vs V991_H and V991_GS vs V991_S group.

### Verification of DEGs by quantitative real-time PCR

**Fig 7:**
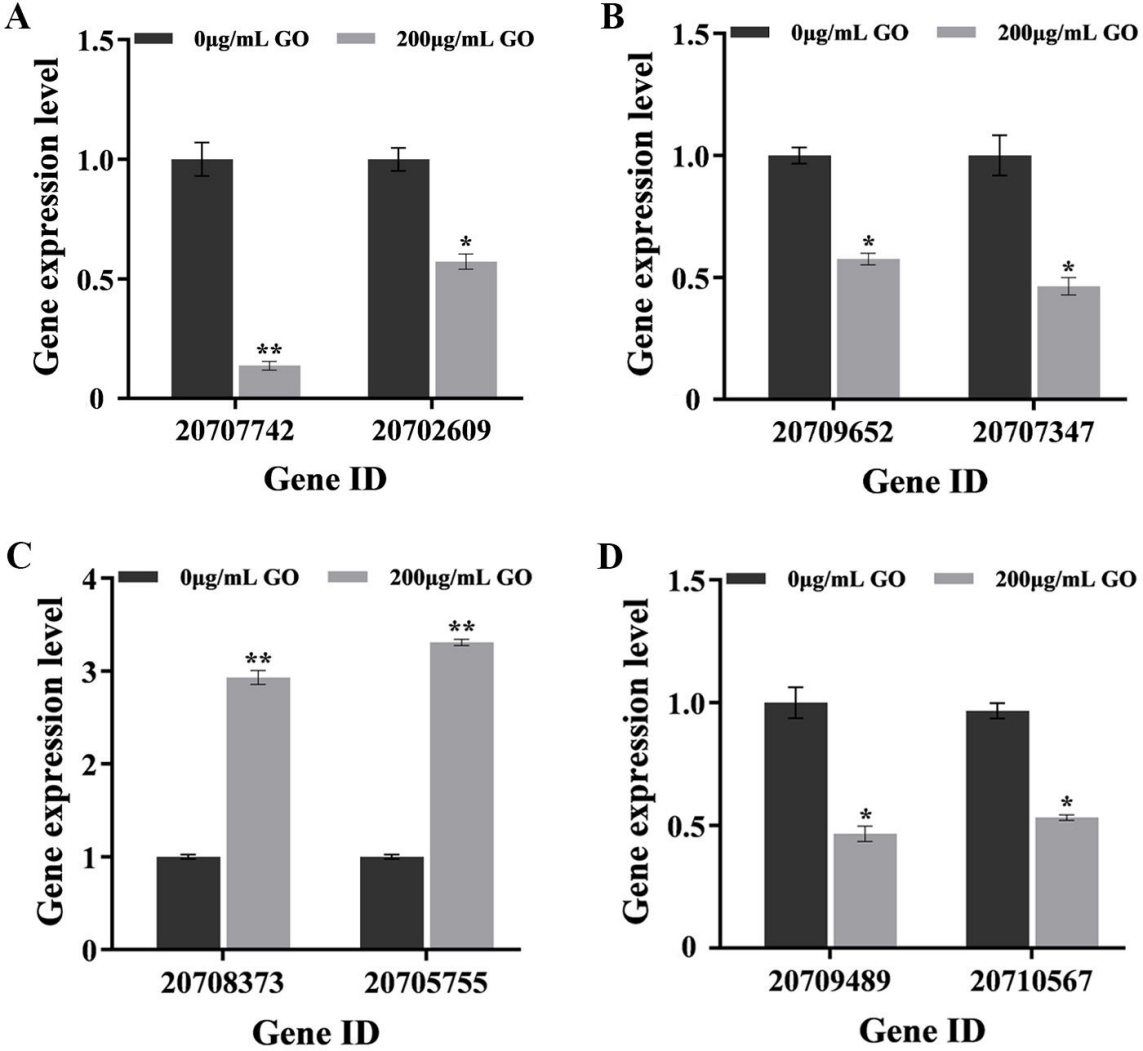
*V. dahliae* gene expression upon GO exposure related to transcription (**A**), carbohydrate metabolic process (**B**), amino acid metabolism, folding, sorting, and degradation (**C**) and ABC transporters (**D**). **P < 0.01, *P < 0.05.

To validate the expression changes of genes, eight DEGs from V991_GH vs V991_H and V991_GS vs V991_S groups enriched in the primary pathways were chosen for qRT-PCR analysis. The gene expression involved in carbohydrate metabolic processes (Fig. 7A), amino acid metabolism (Fig. 7B), and ABC transporters (Fig. 7D) treated with GO was remarkably lower than that in the control group. And the folding, sorting, and degradation of genes encoding chaperone proteins and heat shock proteins were significantly increased with GO treatment (Fig. 7C). The information and expression patterns of these genes in the transcriptome are listed in Table 1. The results demonstrated that the expression patterns of these genes were consistent with the transcriptome data, suggesting the reliability of the transcriptome sequencing results.

**TABLE 1:** RNA-seq analysis of eight genes corresponding to the most enriched pathways

Gene ID	Gene name	Description	Fold change (test/control)
Carbohydrate metabolic process			
20707742 (V991_GS vs V991_S)	VDAG_06279	Beta-glucosidase	0.18 (down)
20702609 (V991_GH vs V991_H)	VDAG_01146	Uncharacterized protein	0.53 (down)
Amino acid metabolism			
20709652 (V991_GS vs V991_S)	VDAG_08189	Glutamate decarboxylase	0.61 (down)
20707347 (V991_GH vs V991_H)	VDAG_05884	Glutamine synthetase	0.53 (down)
Folding, sorting, and degradation			
20708373 (V991_GS vs V991_S)	VDAG_06910	Heat shock protein	1.72 (up)
20705755 (V991_GH vs V991_H)	VDAG_04292	Chaperone protein DNAJ 2	2.21 (up)
ABC transporters			
20709489 (V991_GS vs V991_S)	VDAG_08026	ATP-dependent permease PDR10	0.32 (down)
20710567 (V991_GH vs V991_H)	VDAG_09104	ABC transporter CDR4	0.50 (down)

### GO inhibited fungal infections in cotton

**Fig 8:**
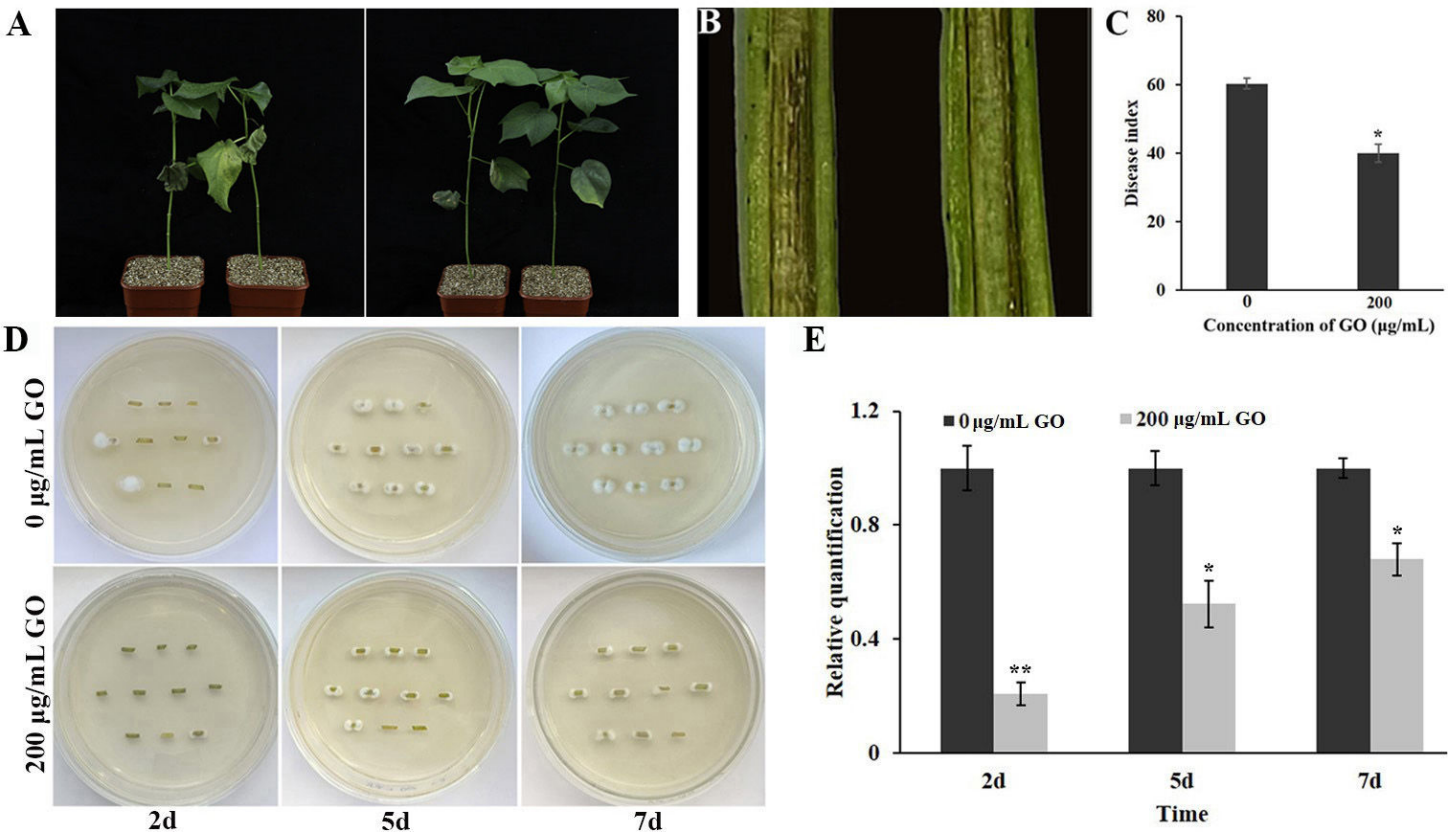
Pathogenicity tests of *V. dahliae* under GO treatment. (**A**) Disease symptoms from control plants and GO-treated plants infected by *V. dahliae*. (**B**) Disease symptoms in infected stems of control and GO-treated plants inoculated with *V. dahliae*. (**C**) Disease index of the control and GO-treated plants at 21 DPI (*n* = 30). (**D**) Stem sections were collected at 2, 5, and 7 DPI, plated on PDA medium, and incubated at 25°C for 7 days. (**E**) qPCR analysis of the relative fungal biomass in stems of the control and GO-treated plants at 2, 5, and 7 days after inoculation with *V. dahliae*.

To investigate the effect of GO on the pathogenicity of *V. dahliae* in cotton, a pot inoculation experiment was conducted. The results showed that cotton plants untreated with GO displayed obvious disease symptoms 21 days post-inoculation (DPI), with many wilted leaves at the bottom (Fig. 8A) and showing brown vascular tissue (Fig. 8B). The disease index of control plants was significantly higher than that of GO-treated plants at 21 DPI (Fig. 8C). Compared to the control plants, GO-treated plants showed lower fungal recovery from stem sections after inoculation with *V. dahliae* (Fig. 8D) and accumulated less fungal biomass (Fig. 8E). These results indicated that GO treatment reduced the pathogenicity of *V. dahliae* in cotton.

## DISCUSSION

*V. dahliae* is one of the extremely pernicious pathogens and invades plant cells through its hyphae produced by a spore (34). It has been proven that GO exerted a prominent inhibitory effect on mycelial growth and spore germination of plant pathogenic fungi, such as *B. sorokiniana* and *P. vitricola* (9, 16). The results of our research revealed that GO treatment can inhibit the growth of fungal mycelia showing a trend of decline in colony diameter and dry weight (Fig. 2B and C). Similarly, the spore germination rate and extension of germ tube were suppressed after GO treatment (Fig. 2E and F). Within the concentration range of 0–200 μg·mL^−1^ GO, the suppressive effect improved with increasing concentration. In addition, in pot experiments, treatment with 200 µg·mL^−1^ GO alleviated the symptoms of Verticillium wilt in cotton (Fig. 8). These findings suggested GO could serve as a prospective and promising alternative to conventional chemical agents or synergistically with fungicides to control Verticillium wilt.

To assess the molecular impact of *V. dahliae* when exposed to GO, a comparative transcriptomics analysis was performed. On this basis, the GO term enrichment and KEGG pathway analysis of the transcriptomic profiles were done. In the downregulated DEGs of V991_GH vs V991_H, as well as V991_GS vs V991_S groups, genes related to the ontology terms “intrinsic component of membrane” and “integral component of membrane” were notably enriched (Fig. 6B and D). Notably, several genes related to cell membrane biosynthesis were found to be downregulated. Among these genes, the gene (20708196) encoding glycerophosphoryl diester phosphodiesterase, which was downregulated by 2.46-fold and 1.75-fold in V991_GH vs V991_H and V991_GS vs V991_S, respectively, is known to play important roles in cell membrane organization (35, 36). Another downregulated gene (20709770) encoding cyclopropane-fatty-acyl-phospholipid synthase (CFA synthase, CFAS) was significantly suppressed by 2.55-fold under GO stress in V991_GH vs V991_H. The lipids containing cyclopropane-fatty-acid (CFA), synthesized by CFAS, safeguard bacteria from unfavorable conditions (37). Sphingolipid, a class of amphoteric lipids containing a sphingosine backbone, is an essential element of biofilm structure, and the sphingoid long-chain base transporter is necessary for sphingolipid synthesis (38). The sphingoid long-chain base transporter gene (20706600) was also found to be downregulated by 4.67-fold in V991_GH vs V991_H. These results indicated that GO impaired biofilm function by downregulating genes involved in biofilm synthesis. It has been demonstrated that membrane damage can lead to alteration of the morphology of the cell and the release of subcellular constituents like nucleic acids and electrolytes (17, 39). For this research, GO treatment can disrupt the cell wall and membrane integrity, which leads to changes in membrane permeability, the morphology of hyphae, and the leakage of contents (Fig. 3). Combined with transcriptional analysis, we speculated that one of the mechanisms of antimicrobial activity of GO on *V. dahliae* is to destroy the cell membrane, consistent with those proposed by previous researches (17, 18, 40). Notably, the downregulated DEGs in the present study differed from those in *Saccharomyces cerevisiae* and *Staphylococcus aureus* under GO treatment (41, 42). This might be attributed to the variation in the composition of the cell membrane, leading to the different antimicrobial impacts of GO on various microorganisms. In addition, a previous study has demonstrated that the cell wall integrity of *F. graminearum* also changed at the molecular level after GO treatment (40). In this work, the chitin synthase gene related to cell wall component synthesis was also downregulated, and chitin content significantly decreased after GO treatment (Fig. 3B), implying the dysfunction of cell wall synthesis occurred by GO.

Lipid peroxidation caused by the oxidation of GO is also considered one of the causes of bacterial membrane damage (21, 39), and many transcriptional level studies have been conducted in the interaction between carbon-derived nanomaterials and microorganisms (43–46). In this work, the gene expression of cytochrome b2, cytochrome P450, NADP-dependent alcohol dehydrogenase, carbonyl reductase, NADPH dehydrogenase, catalase (20710578), catalase-1 (20705124) and catalase-3 (20708038), which are related to oxidative stress response, was downregulated. Catalase (CAT) and superoxide dismutase (SOD), as copper-dependent enzymes, can clear free radicals and protect cells from damage. The overexpression of high-affinity copper transporter genes (20710756, 20707614, and 20708167) implies copper ion starvation of *V. dahliae* after GO treatment (47–49). Combined with the downgraded CAT genes, therefore, we speculated that the ability of SOD and CAT to clear free radicals decreases. Excessive free radicals undergo lipid peroxidation with lipids, ultimately producing MDA. Since MDA is a water-soluble molecule that leaks out when the cell membrane is damaged, the degree of membrane peroxidation can be determined by measuring the MDA content, thereby determining the degree of membrane damage (50, 51). The increase in MDA content in the culture medium reflects oxidative stress, occurring and resulting in cell membrane damage.

Under abiotic stress, fungi could improve their exogenous detoxification capacity and reduce intracellular drug concentration by activating drug efflux transporters to acquire multiple drug resistance (52). Overexpression of ABC transporters and efflux pumps of the major promoter superfamily is considered a major mechanism for fungal drug resistance (53). It was previously reported that genes encoding ABC transporter proteins are directly related to drug resistance (54, 55). In this work, through KEGG pathway analysis, the genes that were downregulated were all concentrated within the pathway of the ABC transporter in V991_GH vs V991_H and in V991_GS vs V991_S. The genes encoding ABC transporter (20710567, 20702834; 20703684, and 20709489) and multidrug resistance protein (20706523, 20702630, 20705930, 20706756, and 20710755) were significantly downregulated in V991_GH vs V991_H or in V991_GS vs V991_S, implying that GO can prevent the development of drug resistance. Tyrosine, as a precursor substance for melanin formation, plays a crucial role in the metabolic pathway of melanin production. Melanin can enhance nutrition, scavenge reactive oxygen species, protect fungi from the harms of environmental stress, promote the development of mycelium, and strengthen their cell wall (56). In this study, the downregulated genes of V991_GH vs V991_H and in V991_GS vs V991_S group were also enriched in tyrosine metabolism, which suggested that GO inhibits tyrosine synthesis, affecting melanin synthesis in *V. dahliae*, thereby impairing the ability of *V. dahliae* to respond to various environmental stresses, ultimately affecting its normal growth. Moreover, the downregulated genes of V991_GH vs V991_H and V991_GS vs V991_S are enriched in nitrogen metabolism (20701552, 20709802, 20701841, 20705768, 20707347, 20705283, 20703780, and 20706586) and starch and sucrose metabolism (20705436, 20708673, 20707742, 20703155, 20704501, 20701871, 20707014, and 20704277) pathways, respectively. Sugar and nitrogen sources are the basic nutritional requirements for fungal growth. Therefore, we speculate that the abnormal regulation of metabolic pathways associated with amino acid and sugar synthesis and breakdown caused by GO is one of the reasons affecting the healthy growth of *V. dahliae*.

Among the upregulated genes of V991_GH vs V991_H, there is a notable enrichment in genes (20710756, 20707614, 20708167, 20711381, and 20708690) related to the GO term “copper ion transmembrane transport activity,” “copper ion homeostasis,” “cellular copper ion homeostasis,” and “transition metal ion transmembrane transport activity” (Fig. 5A). 20710756, 20707614, and 20708167 encode high-affinity copper transporter or high-affinity copper transporter CTR4 responsible for absorbing copper ions under low copper conditions (47–49). And high-affinity copper transporter CTR4 is associated with the expression and pathogenicity of toxic factors in *Cryptococcus* yeast. The △ctr4 mutant strain grows slowly and has significantly reduced pathogenicity (57). The upregulation of 20710756, 20707614, and 20708167 suggests low availability of copper after GO treatment. The content of copper ions in fungi can also affect their absorption of other metal elements, especially iron. The function of upregulated genes (20704918, 20711264, 20705270, 20711268, 20703685, and 20708455) associated with the absorption of iron on the cell surface and the metabolic acclimation to iron-deficient circumstances was also observed, which was different from the upregulated genes from *Saccharomyces cerevisiae* after GO treatment (23). These differences might originate from the disparities in GO concentrations and fungal species. In short, alterations in differential expression of diverse heavy metal transporters could suggest that GO may lead to a disequilibrium in heavy metal homeostasis, subsequently reducing pathogenicity.

### Conclusion

**Fig 9:**
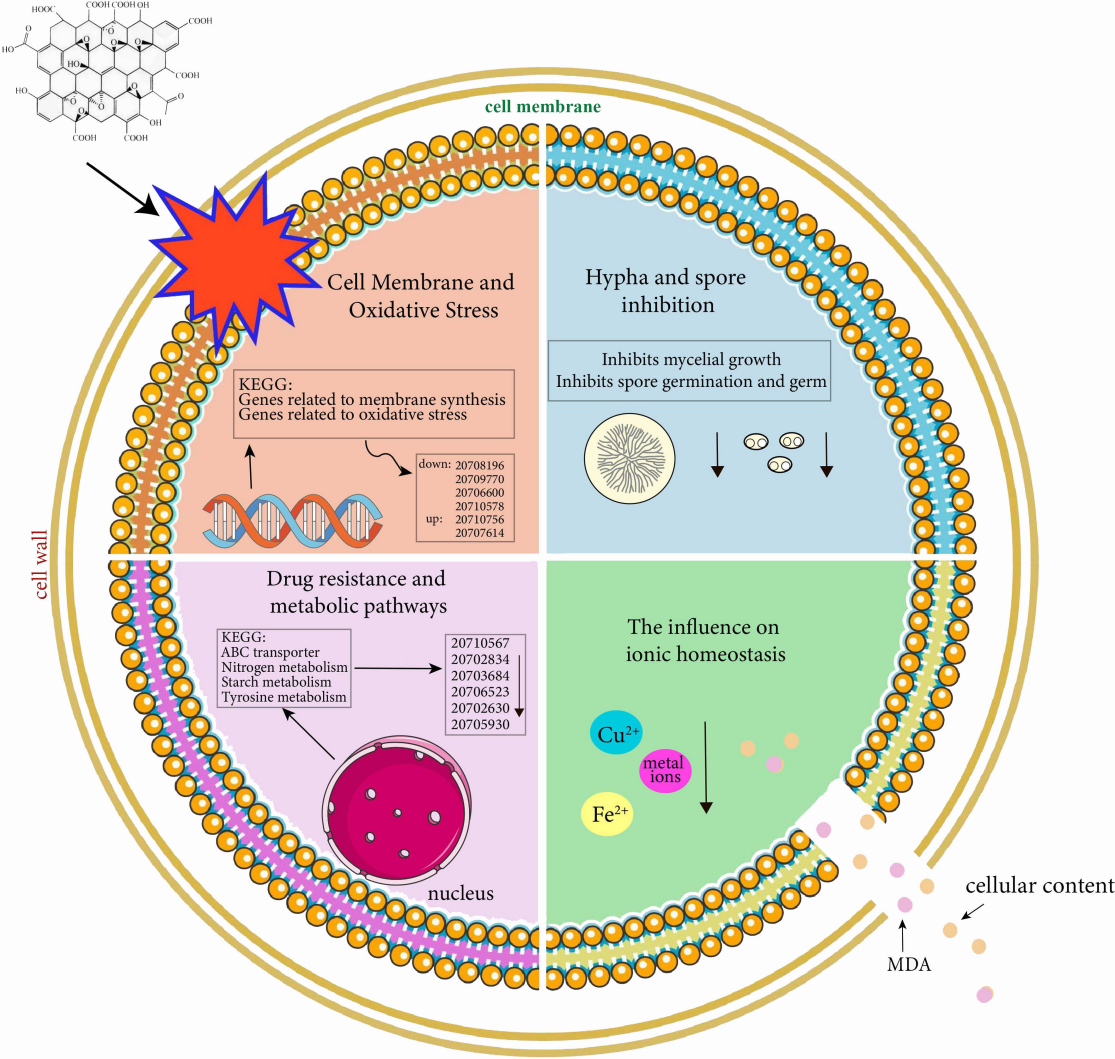
GO can inhibit the mycelial growth and spore germination of *V. dahliae* by destroying the cell membrane to reduce the harm of *V. dahliae* to the host.

In summary, GO could suppress the growth of mycelium and the germination of spores of *V. dahliae*. Transcriptome data display the alteration in a large number of downregulated genes in hyphal elongation and spore germination that are related to intrinsic component and integral component of membrane, and oxidoreductase activity in GO-treated *V. dahliae* (Fig. 9). These phenomena are confirmed by FDA/PI dual color fluorescence experiment, examination of the efflux of cell contents, the cell membrane permeability, and lipid peroxidation after GO treatment. In addition, the application of GO could alleviate the symptoms of cotton wilt infection caused by the pathogen *V. dahliae*. In conclusion, our results provide an experimental basis for the application of GO in Verticillium wilt management.

## Data Availability

The transcriptome data in this article can be found in the NCBI SRA database under accession number PRJNA1172274.

## SUPPLEMENTAL MATERIAL

Table S1 spectrum.01276-25-s0001.docx Primer sequences used in RT-qPCR.

Supplemental material spectrum.01276-25-s0002.docx Tables S2 and S3.

ASM does not own the copyrights to Supplemental Material that may be linked to, or accessed through, an article. The authors have granted ASM a non-exclusive, world-wide license to publish the Supplemental Material files. Please contact the corresponding author directly for reuse.
