# Supplementary material for: In vitro and in vivo inhibitory effects and transcriptional reactions of graphene oxide on Verticillium dahliae
Source: Microbiol Spectr. 2025 Aug 26;13(10):e01276-25. doi: 10.1128/spectrum.01276-25 (PMC12502801; doi:10.1128/spectrum.01276-25)
Supplement: Table S1 — Primer sequences used in RT-qPCR. [file spectrum.01276-25-s0001.docx]

| Supplementary Table 1. The primer sequences were used in RT-qPCR. | |
| --- | --- |
| Name of primer | Sequence of primer 5'-3' |
| qPCR-VDAG06910_20708373-F | TCCAGGCCGACATGAAGCAC |
| qPCR-VDAG06910_20708373-R | CACGCATCTTGACGAGGACCA |
| qPCR-VDAG06279_-20707742F | TTCAGGAGGCTATCTCGTCCAA |
| qPCR-VDAG06279_20707742-R | TGCCAGCTTTAACTGCATCGTAG |
| qPCR-VDAG08026_20709489-F | ATGGACCCTACCAGCAAGTCT |
| qPCR-VDAG08026_20709489-R | GCCGACTTCAAGCTGATACCTT |
| qPCR-VDAG08189_20709652-F | GGTAGTTTTCCGCCTTCGACCA |
| qPCR-VDAG08189_20709652-R | CACTGAAACCCCGCTGCAT |
| qPCR-VDAG05884_20707347-F | ACATTTCCAGGACCGACACT |
| qPCR-VDAG05884_20707347-R | TCGATCCAGATGTACTCGGCCAT |
| qPCR-VDAG04292_20705755-F | AGTACCACCCTGACAAGAACGC |
| qPCR-VDAG04292_20705755-R | CAGCCTCGCCATACTGGTCG |
| qPCR-VDAG09104_20710567-F | CAGCACCTCAACGTCTACGG |
| qPCR-VDAG09104_20710567-R | AAGCTACGCAGAATGTCGAT |
| qPCR-VDAG01146_20702609-F | GCCGCATCGTGTACCTGTCC |
| qPCR-VDAG01146_20702609-R | TCCGACAGAGCGACTCCCC |
| GhUB7-F | GAAGGCATTCCACCTGACCAAC |
| GhUB7-R | CTTGACCTTCTTCTTCTTGTGCTTG |
| ITS1-F | AAAGTTTTAATGGTTCGCTAAGA |
| ST-Ve1-R | CTTGGTCATTTAGAGGAAGTAA |
