# Supplementary material for: In vitro and in vivo inhibitory effects and transcriptional reactions of graphene oxide on Verticillium dahliae
Source: Microbiol Spectr. 2025 Aug 26;13(10):e01276-25. doi: 10.1128/spectrum.01276-25 (PMC12502801; doi:10.1128/spectrum.01276-25)
Supplement: Supplemental material — Tables S2 and S3. [file spectrum.01276-25-s0002.docx]

Supplementary Table 2. Colony diameter after different concentrations of GO treatment.

| GO concentration (μg/mL) | Colony diameter (cm) | | | | |
| --- | --- | --- | --- | --- | --- |
|  | 0 d | 2 d | 4 d | 6 d | 8 d |
| 0 (CK) | 1 | 1.62±0.02a | 2.43±0.04a | 3.55±0.03a | 3.93±0.01a |
| 50 | 1 | 1.53±0.02b | 2.21±0.03b | 2.84±0.04b | 3.22±0.01b |
| 100 | 1 | 1.52±0.02b | 2.14±0.02b | 2.74±0.01c | 3.11±0.03c |
| 200 | 1 | 1.10±0.04c | 2.01±0.03c | 2.42±0.03d | 2.62±0.01d |

Note: Values within the same column followed by different lowercase letters (a, b, and c) are significantly different according to a one-sample t-test (*P* < 0.05).

Supplementary Table 3. Transcriptome data quality control table.

| Sample | Raw  reads | Raw  bases | Clean reads | Clean bases | Error rate (%) | Q20(%) | Q30(%) | GC content (%) | Total  mapped | Multiple mapped | Uniquely  mapped |  |
| --- | --- | --- | --- | --- | --- | --- | --- | --- | --- | --- | --- | --- |
| V991_H1 | 49935070 | 7540195570 | 49434528 | 7431616316 | 0.0119 | 98.77 | 96.18 | 57.59 | 43628119(88.25%) | 502869(1.02%) | 43125250(87.24%) |  |
| V991_H2 | 49740032 | 7510744832 | 49277854 | 7409778107 | 0.0119 | 98.76 | 96.15 | 57.81 | 43244754(87.76%) | 443562(0.9%) | 42801192(86.86%) |  |
| V991_H3 | 46453772 | 7014519572 | 46048482 | 6928945992 | 0.0118 | 98.84 | 96.38 | 58.37 | 42128436(91.49%) | 184308(0.4%) | 41944128(91.09%) |  |
| V991_GH1 | 44021352 | 6647224152 | 43577680 | 6547357312 | 0.0119 | 98.76 | 96.15 | 57.71 | 38751987(88.93%) | 274071(0.63%) | 38477916(88.3%) |  |
| V991_GH2 | 48872834 | 7379797934 | 48385676 | 7267613712 | 0.0119 | 98.77 | 96.16 | 57.89 | 42706039(88.26%) | 340829(0.7%) | 42365210(87.56%) |  |
| V991_GH3 | 41590240 | 6280126240 | 41101306 | 6185096420 | 0.0121 | 98.67 | 95.87 | 57.56 | 35892766(87.33%) | 390082(0.95%) | 35502684(86.38%) |  |
| V991_S1 | 43180204 | 6520210804 | 42759044 | 6428598945 | 0.012 | 98.73 | 95.99 | 57.96 | 38482945(90.0%) | 416028(0.97%) | 38066917(89.03%) |  |
| V991_S2 | 43946736 | 6635957136 | 43494000 | 6539888466 | 0.0119 | 98.75 | 96.14 | 57.93 | 39318888(90.4%) | 348676(0.8%) | 38970212(89.6%) |  |
| V991_S3 | 56119848 | 8474097048 | 55523954 | 8344711948 | 0.012 | 98.74 | 96.08 | 58.13 | 50311361(90.61%) | 460398(0.83%) | 49850963(89.78%) |  |
| V991_GS1 | 50452466 | 7618322366 | 49983898 | 7522127627 | 0.0119 | 98.78 | 96.17 | 57.92 | 45140807(90.31%) | 350607(0.7%) | 44790200(89.61%) |  |
| V991_GS2 | 47002616 | 7097395016 | 46541830 | 7002687614 | 0.0119 | 98.76 | 96.15 | 58.05 | 42407017(91.12%) | 401542(0.86%) | 42005475(90.25%) |  |
| V991_GS3 | 50998412 | 7700760212 | 50514998 | 7597698049 | 0.0119 | 98.8 | 96.29 | 58.01 | 45799353(90.66%) | 480433(0.95%) | 45318920(89.71%) |  |
